# Characterizing reasons for e-cigarette use among Filipino adults who smoke: A cross-sectional study

**DOI:** 10.18332/tpc/221661

**Published:** 2026-07-16

**Authors:** Raniyan Zaman, Lauren Czaplicki, Elizabeth Crespi, Joanna E Cohen, E Ulysses Dorotheo, Madeleine Valera, Ana Mayor, Filomena T Dayagbil, Kevin Welding

**Affiliations:** 1 Johns Hopkins Bloomberg School of Public Health Institute for Global Tobacco ControlBaltimoreUnited States; 2 Southeast Asia Tobacco Control AllianceManilaPhilippines; 3 Vital StrategiesManilaPhilippines; 4 GoodThinking Inc.Pasig CityPhilippines; 5 Cebu Normal University College of Teacher EducationCebuPhilippines

**Keywords:** e-cigarettes, cessation, tobacco control, Philippines

## Abstract

**Introduction:**

There is a potential risk of increased dual use of e-cigarettes and cigarettes among people who currently smoke and use e-cigarettes. Limited data are available on why people who smoke in the Philippines use e-cigarettes, including reasons that relate to smoking cessation. The current study addresses this gap to inform e-cigarette regulatory strategy.

**Methods:**

Data were collected via an online self-administered cross-sectional survey in November 2023 from 611 Filipino adults who smoke and reported current e-cigarette use (i.e. dual users). Participants selected their primary reason for e-cigarette use from a pre-populated list of 15 reasons. We conducted separate logistic regression models of each top reason for e-cigarette use, controlling for demographic and tobacco-use variables.

**Results:**

The top primary reasons for e-cigarette use were to cut down smoking (26%), to help quit smoking (24%), to use when not allowed to smoke cigarettes (11.6%), vaping is less harmful than smoking to others around me (11.6%), and ‘I enjoy the flavor’ (8.5%). Men were more likely than women to select to help quit (adjusted odds ratio, AOR=1.84; p=0.003, 95% CI: 1.22–2.77). Those intending to quit smoking within the year were more likely to select to help quit (AOR=5.22; p<0.001, 95% CI: 2.30–11.9) and to cut down (AOR=2.84, p<0.001, 95% CI: 1.36–5.95), and less likely to select to use when not allowed to smoke (AOR=0.25, p<0.001, 95% CI: 0.13–0.50) and because ‘I enjoy the flavor’ (AOR=0.24, p<0.001, 95% CI: 0.12–0.49).

**Conclusions:**

Some people who smoke in the Philippines use e-cigarettes in order to support smoking cessation; however, there is a risk of prolonged dual use if strong regulations are not implemented to promote complete cessation. Further research should investigate causal factors for e-cigarette use to identify policy pathways for reducing dual use and supporting cessation efforts.

## Introduction

Since the Philippines ratified the World Health Organization Framework Convention on Tobacco Control (WHO FCTC) in 2005, the country has seen progress in reducing adult smoking rates from 28.3% in 2009 to 18.5% in 2021^1^. Part of this decline can be attributed to passing tobacco control laws based on WHO FCTC recommendations, which include raising the price of cigarettes through taxation, restricting smoking in all indoor public places, requiring cigarette packs be sold with graphic health warning labels (HWLs) that cover the bottom 50% of the front and back of the pack, and restricting cigarette marketing in public places and on the internet^2,3^.

In many respects, the Philippines lacks a comprehensive regulatory structure for e-cigarettes, even as data show 6% of adults and 14% of adolescents report ever using an e-cigarette^1,4^. E-cigarette packaging must include 50% HWL coverage, and e-cigarette use is restricted in the same indoor public places as cigarettes^3^. However, public places are permitted to establish ‘designated vaping areas’, and e-cigarettes are taxed at a significantly lower rate. Other marketing restrictions exist to limit the use of flavor descriptors (fruit, candy, or dessert) and imagery (cartoon characters) that are known to appeal to youth, and there is a ban on the sale of e-cigarettes other than plain tobacco or menthol flavored^3,5^. Yet, e-cigarettes are allowed to be marketed and sold online, which may facilitate access and exposure to flavored products^6^.

There is global concern that the sale of flavored e-cigarettes has the potential to increase e-cigarette initiation without improving the likelihood of smoking cessation^7^. Flavored e-cigarettes appeal to youth and nicotine non-consumers^8^, and adolescents are more likely to initiate vaping through flavored e-cigarettes versus non-flavored e-cigarettes^9^. Furthermore, there is evidence that both youth and adults perceive flavored e-cigarettes as less harmful^9,10^ and youth e-cigarette use is associated with uptake of smoking^11-14^. Among those who currently smoke, there is a potential risk of increased dual use of cigarettes and e-cigarettes^15^. While some studies suggest that e-cigarettes may contribute to smoking cessation if used exclusively^16^, people who use both cigarettes and e-cigarettes in an attempt to quit smoking often struggle to fully make the transition to sole e-cigarette use, which results in prolonged dual use of both products^17^. Rather than facilitating smoking cessation, under-regulated e-cigarette markets may entrench dual use^18^ – prolonging nicotine dependence and undermining smoke-free policies, particularly when vaping is permitted in places where smoking is banned^19^.

To date, very limited information is available on why Filipino adults who smoke use e-cigarettes. In other countries, like the US, adults who smoke and use e-cigarettes commonly cite smoking cessation, convenience, and other health-related factors among their reasons for e-cigarette use^20^. One study from Malaysia found that common reasons for e-cigarette use among people who smoke included reducing the number of cigarettes smoked, a pleasant taste, to quit smoking, and for their enjoyment^21^. The current research fills a gap in exploring the main reasons for e-cigarette use among adults who smoke in under-regulated e-cigarette environments in low-and middle-income countries, such as the Philippines.

## Methods

### Study sample

Data were collected in November 2023 via an online survey. The Manila-based market research firm, GoodThinking, managed data collection. Participants were recruited through the online panel PureProfile. Panelists were eligible if they were at least 18 years old, lived in the Philippines, smoked at least 100 cigarettes in their lifetime, smoked on at least one of the past 30 days, reported buying their own cigarettes, and were able to speak and read at least one of the languages that the survey was offered in (English, Tagalog, or Cebuano).

In total, 911 adults who smoke completed the survey. We removed 25 participants (2.7% of the sample) who completed the survey in under 4 min, or roughly one standard deviation below the average completion time (~10 min), resulting in 886 respondents. We restricted the sample to the 618 adults who both currently smoke (defined as those who smoked 100 cigarettes in their lifetime and reported smoking in the past 30 days) and reported using an e-cigarette or vaping device within the past 30 days. Our final analytic sample included 611 participants who had complete data. We removed seven participants (~ 1%) from the analysis; six participants did not report household income, and one did not report time to first cigarette.

The distribution of our sample of Filipino adults who smoke was similar to the latest Global Adult Tobacco Survey (GATS) estimates in terms of age and wealth/income; however, women who smoke were over-represented compared to GATS data^1^. Additionally, past-day e-cigarette use in our sample of people who smoke was substantial (n=618/886 or roughly 70%) and markedly higher than the past 30 day point prevalence of e-cigarette use among the general population in the Philippines (regardless of smoking status)^1,2^. However, the prevalence of e-cigarette use among people who smoke from a nationally representative surveillance survey is not available to make a direct comparison of how estimates in this study might differ.

All participants provided digitally written consent to participate. The study protocol was approved by the Johns Hopkins Bloomberg School of Public Health Institutional Review Board (#00024497) and the Philippine Social Science Council-Social Science Ethics Review Board (CF-23–24).

### Demographic measures

Participants provided information on their age, gender (male, female), and household income (PHP) (<40000, 40000–59999, 60000–99999, 100000–149999, 150000–199999, 200000–249999, 250000–299999, 300000–349999, 350000–399999, 400000–449999, 450000–499999, ≥500000, and No income). For age, we categorized participants into the following age groups: 18–24, 25–34, and ≥35 years. For personal income, we categorized income (PHP) into the following categories: <60000 (including those who reported ‘No income’), 60000–299999, and ≥300000.

### E-cigarette-related measures

Participants reported frequency of e-cigarette use (less than weekly, 1–3 days per week, 4–7 days per week), most recent device type used (reusable, disposable), and most recent flavor used (tobacco, menthol, mint, fruit, dessert, some other flavor). The last flavor used was dichotomized into tobacco/menthol or other. Dichotomizing e-cigarette flavors in this fashion allowed us to run a stable model, given the low distribution of tobacco flavor use, and allowed us to compare the most commonly available flavors for cigarettes (tobacco, menthol) versus other flavors. Participants were provided with a definition of device type where reusable was defined as ‘you recharge the device when the battery life is low or at 0% AND you refill or replace the tank/pod/cartridge when it runs out of e-liquid’) and disposable was defined as ‘you discard the entire device when the battery life is low or at 0% OR when the device runs out of e-liquid’.

### Smoking-related measures

We calculated smoking dependence level (score: low 0–1, medium 2–4, high 5–6) by summing participants’ responses to two items: time to first cigarette (≤5 min [3 points], 6–30 min [2 points], 31–60 min [1 point], >60 min [0 points]) and number of cigarettes smoked each day (≤10 [0 points], 11–20 [1 point], 21–30 [2 points], ≥31 [3 points])^22^. Participants were also asked about their intention or plan for quitting smoking with response options: ‘I plan to quit in 1 month’, ‘I plan to quit in 6 months’, ‘I plan to quit in 12 months’, ‘I plan to quit someday but not in the next 12 months’, ‘I am not interested in quitting’, and ‘I don’t know’. We divided this variable into three categories: 1) combining responses related to quitting within 1 month, 6 months, and 12 months; 2) planning to quit someday but not in the next 12 months; and 3) combining responses ‘I don’t know’ and ‘I am not interested in quitting’ to create a category that reflected no interest or uncertain interest in quitting.

### Outcome measure

Participants were asked: ‘What is your PRIMARY reason for using an e-cigarette or vaping device?’, select one answer only. Response options were informed by prior research^20,23^ and included the following (in the order presented to participants): ‘To help quit smoking cigarettes’, ‘To cut down smoking cigarettes’, ‘To use when I am not allowed to smoke cigarettes’, ‘Vaping is less harmful than others around me’, ‘Absence of smell’, ‘To socialize’, ‘To have a longer break at work’, ‘I enjoy the flavor’, ‘I enjoy the boost’, ‘I am addicted’, ‘My friends use them’, ‘Curiosity/just wanted to try them’, ‘Stress, anxiety, depression, or other mental health concerns’, ‘Some other reason (open-ended response)’, and ‘Prefer not to answer’. Response options were presented to participants in the order listed above, which broadly followed the order of response options from a prior study^24,25^. The reasons ‘To help quit smoking cigarettes’ and ‘To cut down smoking cigarettes’ are both directly related to cessation but conceptually distinct. The former can be interpreted as an intention to fully eliminate smoking, which would immediately reduce smoke exposure, while the latter can be interpreted as a step toward quitting that would still lead to regular smoke exposure.

### Statistical analysis

We used Stata (version BE 19.5) to descriptively characterize the study sample, including the distribution of reasons for e-cigarette use. For each of the top reasons for use, which we defined as a reason selected by at least 5% of the sample, we conducted individual logistic regressions of selecting (vs not selecting) that statement (e.g. ‘to help quit smoking’) as the primary reason for e-cigarette use. Models were adjusted for the following factors that are often related to and possible confounders of e-cigarette use and reasons for e-cigarette use, including demographic measures (age, gender, and income)^21,26,27^, e-cigarette-related behavioral measures (last device type used, last flavor used, frequency of use), and smoking-related behavioral measures (smoking dependence level, intention to quit). Crude odds ratios for each model are provided in Supplementary file Table 1 to facilitate comparison to the adjusted estimates.

Given the large proportion of women who smoke in our sample, we stratified the final models by gender to determine if there were any key differences in the pattern of results. Significance level was set to 0.01 to account for multiple comparisons (p=0.05/5 models).

## Results

Approximately 70% of the study sample (n=618/886 Filipino adults who smoked) reported e-cigarette use in the past 30 days. Table 1 presents the demographic characteristics and tobacco use behaviors of the analytic sample of 611 Filipino adults who reported smoking and using e-cigarettes. Approximately 44% were aged 25–34 years, followed by 29% who were 18–24 years, and 27% who were ≥35 years. Over half were men (57.1%), and 42.9% reported an annual income (PHP) between 60000–300000 versus <60000 (35.5%) or ≥300000 (28.8%). Most (70.2%) had most recently used a reusable e-cigarette device compared to a disposable device (29.8%). With respect to flavor, 4.6% reported tobacco as the last flavor they used, 36.8% reported menthol, and the remaining 58.6% reported some other flavor (31.4% fruit, 17.5% mint, and 8.5% dessert). Few participants (3.1%) reported less than weekly e-cigarette use, and most reported using e-cigarettes 1–3 days per week (48.6%) or 4–7 days per week (48.3%). In terms of smoking dependence, over half (61.4%) of participants reported medium dependence, and around one-third (35.3%) reported low dependence. Most participants (64.8%) reported an intention to quit smoking within a year, and 22.7% reported an intention to quit someday but not within the next year. The top five reported primary reasons for e-cigarette use were: to cut down smoking cigarettes (26.3%), to help quit smoking cigarettes (24.7%), to use when not allowed to smoke cigarettes (11.6%), the belief that vaping is less harmful than smoking to others around me (11.6%), and enjoyed the flavor (8.5%). The remaining primary reasons for e-cigarette use were selected by <5% of the sample.

**Table 1:** Demographic characteristics and tobacco use behaviors of Filipino adults who smoke and reported e-cigarette use within the past 30 days, November 2023 (N=611)

Characteristics	% (n)
**Age** (years)	
18–24	28.6 (175)
25–34	44.5 (272)
≥35	26.8 (164)
**Gender**	
Female	42.9 (262)
Male	57.1 (349)
**Income** (PHP)	
>60000	35.7 (218)
60000–299999	35.5 (217)
≥300000	28.8 (176)
**E-cigarette device type**	
Reusable	70.2 (429)
Disposable	29.8 (182)
**Last e-cigarette flavor used**	
Tobacco	4.6 (28)
Menthol	36.8 (225)
Other	58.6 (358)
**Frequency of e-cigarette use**	
Do not use weekly	3.1 (19)
1–3 days per week	48.6 (297)
4–7 days per week	48.3 (295)
**Smoking dependence level**	
Low	35.3 (216)
Medium	61.4 (375)
High	3.3 (20)
**Intention to quit smoking**	
Not interested/don’t know	12.4 (76)
Intend to quit within a year	64.8 (396)
Intend to quit someday (>1 year)	22.7 (139)
**Primary reason for e-cigarette use**	
To help quit smoking cigarettes	24.7 (151)
To cut down smoking cigarettes	26.3 (161)
To use when I am not allowed to smoke cigarettes	11.6 (71)
Vaping is less harmful than smoking to others around me	11.6 (71)
Absence of smell	4.1 (25)
To socialize	2.0 (12)
To have a longer break at work	1.3 (8)
I enjoy the flavor	8.5 (52)
I enjoy the boost	0.8 (5)
I am addicted	0.8 (5)
My friends use them	1.5 (9)
Curiosity/just wanted to try them	2.8 (17)
Stress/anxiety/depression/other mental health concerns	3.4 (21)
Some other reason	0.2 (1)
Prefer not to answer	0.3 (2)

PHP: 1000 Philippine Pesos about US$16.

Table 2 presents the results from the logistic regressions. Participants who reported intending to quit smoking within a year had significantly higher odds of selecting ‘to cut down smoking cigarettes’ as their primary reason for e-cigarette use if they intended to quit smoking within a year (adjusted odds ratio, AOR=2.84; 95% CI: 1.36–5.95, p=0.006) or intended to quit someday but not within the next year (AOR=2.93; 95% CI: 1.31–6.52, p=0.009) versus not being interested in/not knowing if they want to quit. Participants had significantly higher odds of selecting ‘to help quit smoking’ as their primary reason for e-cigarette use if they were men (AOR=1.84; 95% CI: 1.22–2.77, p=0.003) versus women and if they reported intending to quit within a year (AOR=5.22; 95% CI: 2.30–11.9, p<0.001) versus no clear intention to quit. Participants had significantly lower odds of selecting ‘to use when I am not allowed to smoke cigarettes’ (AOR=0.25; 95% CI: 0.13–0.50, p<0.001) and ‘I enjoy the flavor’ (AOR=0.24; 95% CI: 0.12–0.49, p<0.001) as their primary reason for e-cigarette use, respectively, if they intended to quit within the next year versus no clear intention to quit smoking. There was no significant association between selecting ‘vaping is less harmful than smoking to others around me’ and the demographic or tobacco use variables examined in the regression model.

**Table 2:** Adjusted logistic regression models of choosing (vs not choosing) one of the top five reasons for e-cigarette use of Filipino adults who smoke and reported e-cigarette use within the past 30 days, November 2023 (N=611)

Variables	To cut down smoking cigarettes	To help quit smoking cigarettes	To use when not allowed to smoke cigarettes	Vaping is less harmful than smoking to others	I enjoy the flavor
	AOR (95% CI)	AOR (95% CI)	AOR (95% CI)	AOR (95% CI)	AOR (95% CI)
**Age** (years)					
18–24 (ref.)	1	1	1	1	1
25–34	1.16 (0.72–1.86)	1.23 (0.76–1.99)	0.71 (0.38–1.34)	0.66 (0.36–1.20)	0.76 (0.37–1.56)
≥35	1.28 (0.76–2.17)	1.01 (0.58–1.76)	0.64 (0.30–1.34)	0.83 (0.41–1.65)	0.94 (0.42–2.12)
**Gender**					
Female (ref.)	1	1	1	1	1
Male	1.16 (0.79–1.70)	1.84 (1.22–2.77)**	0.58 (0.34–0.99)*	0.67 (0.40–1.11)	0.84 (0.46–1.53)
**Income** (PHP)					
>60000 (ref.)	1	1	1	1	1
60000–299999	1.22 (0.77–1.94)	1.11 (0.70–1.77)	1.45 (0.77–2.75)	1.12 (0.62–2.04)	0.83 (0.40–1.72)
≥300000	1.80 (1.11–2.91)*	0.75 (0.44–1.25)	1.21 (0.60–2.43)	0.82 (0.41–1.63)	1.03 (0.48–2.19)
**E-cigarette device type**					
Reusable (ref.)	1	1	1	1	1
Disposable	1.14 (0.76–1.72)	1.36 (0.89–2.08)	0.68 (0.37–1.27)	1.04 (0.59–1.80)	0.40 (0.18–0.88)*
**Last e-cigarette flavor used**					
Tobacco/menthol (ref.)	1	1	1	1	1
Other	1.22 (0.83–1.79)	0.77 (0.51–1.14)	1.04 (0.61–1.77)	1.09 (0.65–1.83)	1.02 (0.56–1.86)
**Frequency of e-cigarette use**					
Do not use weekly	0.32 (0.07–1.44)	0.43 (0.09–1.98)	1.61 (0.42–6.22)	0.32 (0.04–2.47)	1.46 (0.30–7.07)
1–3 days per week	1.26 (0.86–1.84)	0.71 (0.47–1.05)	1.55 (0.90–2.66)	0.76 (0.45–1.27)	1.14 (0.62–2.09)
4–7 days per week (ref.)	1	1	1	1	1
**Smoking dependence level**					
Low (ref.)	1	1	1	1	1
Medium	0.80 (0.54–1.19)	1.39 (0.90–2.14)	2.05 (1.12–3.76)*	0.87 (0.51–1.47)	0.61 (0.33–1.14)
High	0.41 (0.11–1.51)	1.95 (0.66–5.79)	4.60 (1.39–15.2)*	0.34 (0.04–2.68)	1.21 (0.30–4.76)
**Intention to quit smoking**					
Not interested/don’t know (ref.)	1	1	1	1	1
Intend to quit within a year	2.84 (1.36–5.95)**	5.22 (2.30–11.9)***	0.25 (0.13–0.50)***	0.61 (0.30–1.23)	0.24 (0.12–0.49)***
Intend to quit someday (>1 year)	2.93 (1.31–6.52)**	1.32 (0.51–3.43)	0.87 (0.42–1.79)	0.69 (0.30–1.58)	0.35 (0.15–08.1)*

PHP: 1000 Philippine Pesos about US$16.

* p<0.05

** p<0.01

*** p<0.001.

AOR: adjusted odds ratio.

Supplementary file Tables 2A and 2B present the results from the models when stratified by gender. When restricted to respondents identifying as men, findings broadly follow the same pattern as the full sample. One difference was that men with high smoking dependence had higher odds (AOR=10.2; 95% CI: 1.99–52.7, p<0.01) of selecting ‘to use when not allowed to smoke’ compared to men with low smoking dependence. When we restricted to respondents identifying as women, we found that there were no significant associations between intention to quit smoking and the outcomes. We also found that women who reported a household income of ≥300000 PHP, had higher odds (AOR=3.31; 95% CI: 1.52–7.19, p<0.01) of selecting ‘to cut down smoking cigarettes’ than women with incomes below <60000 PHP.

## Discussion

This is one of the first studies to investigate reasons for e-cigarette use among Filipino adults who smoke. We found that in our sample, the top reasons for e-cigarette use among people who smoke were to cut down or to help quit smoking, where about a quarter of people in this sample reported either one of these two reasons. A smaller but notable number of participants reported their primary reason for using e-cigarettes was because they believed vaping was less harmful than smoking to others, or they used e-cigarettes when they were not allowed to smoke.

These findings broadly align with research in Malaysia and the US, where the top reasons for e-cigarette use among people who smoke were to cut down or quit smoking^20,21^, and with a US study where people who smoke and use e-cigarettes often cited consideration of others and convenience as one of the top reasons for e-cigarette use^20^. This study suggests that people who smoke in the Philippines are using e-cigarettes for similar reasons compared to people who smoke in other countries, and when asked to select their top reason for use, respondents report motivations for use that are largely related to personal smoking cessation/reduction goals.

In this study, less than a tenth of the sample reported that enjoyment from e-cigarette flavors was their primary reason for e-cigarette use. Other studies from Malaysia and the US conducted among adults who smoke and use e-cigarettes found that a higher proportion of respondents commonly cite flavor or taste as one of their reasons for use when asked to select from a list of options^20,21^. It is possible that participants in our study would have endorsed flavor if the question allowed them to select multiple reasons for use. However, when forced to choose a single reason, flavors seemed to be rated as less important than other reasons like quitting or reducing their smoking. This data could lend weight to support a broader ban on e-cigarette non-tobacco flavors, which are known to appeal to youth and are often cited as a reason for e-cigarette use initiation among people who do not smoke^28,29^, particularly if they may not be necessary to support smoking cessation motivations for e-cigarette use among people who use both products.

We found intention to quit smoking within 1 year was significantly associated with greater odds of selecting ‘to cut down smoking’ or ‘to help quit smoking’ as the primary reason for e-cigarette use among participants in this study. Intention to quit smoking is an important precursor to quitting^30^, and our results suggest notable e-cigarette use among Filipino adults who smoke and indicate readiness to quit smoking. While it is possible that study participants who indicated their primary reason for e-cigarette use was to help quit smoking may be more primed to quit smoking compared to those who indicated their primary reason for e-cigarette use was to reduce smoking; both groups are at risk of prolonged dual use if they do not transition to exclusive e-cigarette use. Further, longitudinal studies of people who smoke and use e-cigarettes often find that this population rarely successfully transitions to exclusive e-cigarette use^17^. Therefore, our data can only provide insight into the motivations of people who use cigarettes and e-cigarettes and cannot address whether e-cigarettes are an effective smoking cessation tool among the study population. Further, those participants who did not intend to quit smoking or were uncertain about quitting were more likely to select using e-cigarettes when they were not allowed to smoke, and enjoyed flavors as their primary reason for use. These data provide insight into how dual use of cigarettes and e-cigarettes may continue if gaps in policy are not closed.

Overall, we did not observe many differences in selecting one of the top five reasons for using e-cigarettes by age or income. We did observe that individuals who selected ‘to help quit smoking’ as their primary reason for e-cigarette use were more likely to be men than women. There is a large gender disparity with respect to smoking in the Philippines, where approximately ten times more men than women report smoking tobacco daily^1^. In the supplemental analysis, we found that men with a higher smoking dependence were more likely to select ‘to use when not allowed to smoke’ as their primary reason for e-cigarette use compared to men with a lower smoking dependence. This pattern highlights the complex interplay between dual use, smoking dependence, and use of e-cigarettes when not allowed to smoke, particularly for men who smoke in the Philippines. However, the use of e-cigarettes when not allowed to smoke might also sustain nicotine dependence and undermine the health benefits of smoke-free regulations. This data adds to the evidence base that can be used to consider policy pathways to strengthen indoor air policies to reduce overall nicotine intake.

We did not observe any significant differences in selecting a reason for e-cigarette use with the last e-cigarette flavor or device type used. It is possible that device features are not related to intrinsic motivations related to quitting smoking or reducing smoke exposure to others, and participants may be selecting devices and flavors based on other factors related to availability or cost. We also did not observe significant relationships with our outcomes and frequency of e-cigarette use or smoking dependence level. There was a notable (though not statistically significant) relationship between higher levels of smoking dependence and greater odds of selecting the ability to use an e-cigarette when smoking is not allowed as the primary reason for e-cigarette use.

### Limitations

This study is subject to several limitations. First, although participants could provide a write-in response for an unlisted primary reason for use, our response options may not be exhaustive of reasons for use (e.g. cost/affordability). Participants were also instructed to select only one primary reason, which may not reflect people who had multiple reasons for e-cigarette use. Additionally, the order of response options for the primary reason for e-cigarette use was not randomized in the online survey. It is possible that primacy bias toward selecting the first options on the list influenced response rate. Second, although our study utilizes a sample of people across three regions of the Philippines who smoke, this was not a nationally representative sample of Filipino adults who smoke and may not be reflective of the experience of all individuals who smoke and use e-cigarettes in the Philippines. Women, in particular, were over-represented compared to GATS estimates^1^. Additionally, our data are cross-sectional, and we cannot establish a temporal relationship between reasons for e-cigarette use and quit intention or account for how reasons for e-cigarette use may have changed over time or in relation to when e-cigarette use started. Our study design also does not allow us to follow individuals over time to assess whether citing a cessation-related reason for e-cigarette use will contribute to smoking cessation. Smoking and e-cigarette use status are self-reported only and were not biochemically verified, leaving our data subject to misclassification bias. There is also a possibility of residual confounding from unmeasured variables not included in the analysis.

## Conclusions

This study provides emerging insights into the reasons why Filipino adults who smoke use e-cigarettes. Our findings indicate that around half of the study sample cited smoking cessation-related reasons as their primary reason for e-cigarette use; however, others are primarily using e-cigarettes when they are not allowed to smoke or they enjoy the flavors. These data provide insight into the motivations for people who smoke and use e-cigarettes, but not into whether e-cigarettes are an effective cessation aid. Further research, along with our data, could support strengthening the Philippines’ e-cigarette regulatory approach by limiting the appeal of e-cigarettes among people who do not use tobacco while also creating conditions that optimize the potential for complete smoking cessation among people who currently smoke and use e-cigarettes in the Philippines. Such measures could contribute to reducing smoking rates in the country and region, and offer insights for other countries facing similar e-cigarette-related regulatory challenges.
