# Supplementary material for: Characterizing reasons for e-cigarette use among Filipino adults who smoke: A cross-sectional study
Source: Tob Prev Cessat. 2026 Jul 16;12:10.18332/tpc/221661. doi: 10.18332/tpc/221661 (PMC13386058; doi:10.18332/tpc/221661)
Supplement: Supplementary file Material 1. [file TPC-12-44-s001.pdf]

TOBACCO PREVENTION AND CESSATION

Supplementary file

© 2026 Czaplicki L. et al.

DOI:
10.18332/tpc/221661

The content has been provided by the author(s) and has not been reviewed, verified, or endorsed by European Publishing. It may not have undergone peer review. The views, opinions, and recommendations expressed are solely those of the author(s) and do not necessarily reflect the position of European Publishing. European Publishing accepts no responsibility or liability for any consequences arising from the use of, or reliance on, this content.

Supplemental Table 1. Crude logistic regression models of choosing (vs. not choosing) one of the top five reasons for e-cigarette use from a cross-sectional survey of Filipino adults who smoke and reported e-cigarette use within the past 30 days (n=611) conducted in November 2023.

	**To cut down smoking cigarettes**	**To help quit smoking cigarettes**	**To use when not allowed to smoke cigarettes**	**Because vaping is less harmful than smoking to others**	**Because I enjoy the flavor**
	OR (95% CI)	OR (95% CI)	OR (95% CI)	OR (95% CI)	OR (95% CI)
**Age**					
18 to 24 years old	ref	ref	ref	ref	ref
25-34 years old	1.25 (0.80-1.96)	1.14 (0.73-1.77)	0.93 (0.52-1.56)	0.63 (0.35-1.12)	0.83 (0.42-1.64)
≥ 35 years old	1.53 (0.93-2.49)	0.92 (0.56-1.53)	0.80 (0.41-1.57)	071 (0.37-1.34)	1.00 (0.48-2.09)
**Gender**					
Women	ref	ref	ref	ref	ref
Men	1.11 (0.77-1.60)	1.79 (1.22-2.64)**	0.65 (0.40-1.08)	0.65 (0.40-1.08)	0.79 (0.45-1.40)
**Income (in PHP)**					
> 60,000	ref	ref	ref	ref	ref
60,000-299,999	1.23 (0.79-1.93)	1.05 (0.69-1.62)	1.25 (0.70-2.26)	1.09 (0.62-1.93)	0.89 (0.45-1.76)
≥ 300,000	1.83 (1.17-2.87)**	0.77 (0.48-1.23)	1.09 (0.57-2.05)	0.79 (0.41-1.51)	1.05 (0.52-2.10)
**E-cigarette device type**					
Reusable	ref	ref	ref	ref	ref
Disposable	1.12 (0.76-1.66)	1.28 (0.86-1.90)	0.65 (0.36-1.17)	1.07 (0.62-1.82)	0.40 (0.18-0.87)*
**Last e-cigarette flavor used**					
Tobacco/menthol	ref	ref	ref	ref	ref
All other flavors	1.31 (0.90-1.89)	0.79 (0.55-1.15)	0.90 (0.55-1.48)	1.10 (0.66-1.82)	0.88 (0.50-1.56)
**Frequency of e-cigarette use**					
Do not use weekly	0.36 (0.08-1.58)	0.30 (0.07-1.35)	1.65 (0.46-6.01)	0.36 (0.05-2.81)	1.32 (0.29-6.09)
1-3 days per week	1.24 (0.86-1.78)	0.76 (0.52-1.10)	1.29 (0.78-2.15)	0.76 (0.46-1.26)	1.08 (0.61-1.93)
4-7 days per week	ref	ref	ref	ref	ref
**Smoking Dependence Level**					
Low	ref	ref	ref	ref	ref
Medium	0.85 (0.59-1.24)	1.50 (1.00-2.25)	1.61 (0.91-2,85)	0.79 (0.48-1.31)	0.62 (0.35-1.12)
High	0.44 (0.12-1.55)	1.72 (0.63-4.74)	3.66 (1.19-11.2)*	0.34 (0.04-2.63)	1.48 (0.40-5.44)
**Intention to quit smoking**					
Not interested/don't know	ref	ref	ref	ref	ref
Intend to quit within a year	2.97 (1.43-6.16)**	4.71 (2.10-10.5)***	0.26 (0.13-0.51)***	0.65 (0.32-1.30)	0.24 (0.12-0.48)***
Intend to quit someday (> 1 year)	2.90 (1.32-6.38)**	1.28 (0.50-3.26)	0.80 (0.40-1.59)	0.69 (0.31-1.55)	0.35 (0.15-0.79)*

Note: OR=odds ratio; CI=confidence interval; ref=reference group; PHP=Philippine peso
* p<0.05, **p<0.01, ***p<0.001

Supplemental Table 2.A Adjusted logistic regression models of choosing (vs. not choosing) one of the top five reasons for e-cigarette use from a cross-sectional survey of adult Filipino men who smoke and reported e-cigarette use within the past 30 days conducted in November 2023.

	**To cut down smoking cigarettes** (n=337)	**To help quit smoking cigarettes** (n=337)	**To use when not allowed to smoke cigarettes** (n=349)	**Because vaping is less harmful than smoking to others** (n=337)	**Because I enjoy the flavor** (n=349)
	aOR (95% CI)	aOR (95% CI)	aOR (95% CI)	aOR (95% CI)	aOR (95% CI)
**Age**					
18 to 24 years old	ref	ref	ref	ref	ref
25-34 years old	1.50 (0.77-2.90)	1.16 (0.62-2.16)	0.54 (0.21-1.36)	0.45 (0.18-1.13)	0.47 (0.16-1.35)
≥ 35 years old	1.77 (0.86-3.64)	0.90 (0.44-1.82)	0.36 (0.12-1.06)	1.02 (0.40-2.61)	0.50 (0.15-1.64)
**Income (in PHP)**					
> 60,000	ref	ref	ref	ref	ref
60,000-299,999	1.13 (0.62-2.06)	0.91 (0.50-1.65)	1.97 (0.77-5.04)	0.96 (0.40-2.32)	1.01 (0.35-2.94)
≥ 300,000	1.21 (0.64-2.29)	0.85 (0.45-1.61)	2.31 (0.85-6.32)	0.91 (0.34-2.38)	1.76 (0.60-5.14)
**E-cigarette device type**					
Reusable	ref	ref	ref	ref	ref
Disposable	1.22 (0.72-2.07)	1.46 (0.85-2.49)	1.23 (0.54-2.80)	0.61 (0.26-1.45)	0.38 (0.12-1.20)
**Last e-cigarette flavor used**					
Tobacco/menthol	ref	ref	ref	ref	ref
All other flavors	1.15 (0.69-1.92)	0.76 (0.46-1.26)	0.99 (0.45-2.19)	0.78 (0.37-1.64)	0.60 (0.25-1.44)
**Frequency of e-cigarette use**					
Do not use weekly	empty	empty	1.83 (0.31-10.8)	0.50 (0.06-4.33)	1.09 (0.11-10.5)
1-3 days per week	1.20 (0.73-1.99)	0.69 (0.42-1.15)	1.88 (0.85-4.17)	0.63 (0.29-1.37)	1.32 (0.55-3.17)
4-7 days per week	ref	ref	ref	ref	ref
**Smoking Dependence Level**					
Low	ref	ref	ref	ref	ref
Medium	0.67 (0.39-1.13)	1.49 (0.85-2.61)	1.95 (0.75-5.10)	0.76 (0.35-1.64)	0.71 (0.28-1.79)
High	0.39 (0.07-2.04)	1.36 (0.30-6.08)	10.2 (1.99-52.7)**	empty	2.58 (0.47-14.1)
**Intention to quit smoking**					
Not interested/don't know	ref	ref	ref	ref	ref
Intend to quit within a year	3.77 (1.28-11.1)*	4.82 (1.80-12.9)**	0.19 (0.07-0.49)**	0.49 (0.18-1.28)	0.15 (0.05-0.39)***
Intend to quit someday (> 1 year)	4.80 (1.52-15.1)**	1.33 (0.43-4.15)	0.47 (0.17-1.32)	0.57 (0.18-1.77)	0.28 (0.09-0.89)*

Note: aOR=adjusted odds ratio; CI=confidence interval; ref=reference group; PHP=Philippine peso; empty=there was no variation in selecting or not selecting the reason for use for this variable category and the model failed to provide a reliable estimate; the statistical software automatically dropped the observations that 'perfectly' predicted the outcome resulting in lower n's for some models.
* p<0.05, **p<0.01, ***p<0.001; Significance level set at p<0.01 (or 0.05/5 comparisons)

Supplemental Table 2.B Adjusted logistic regression models of choosing (vs. not choosing) one of the top five reasons for e-cigarette use from a cross-sectional survey of adult Filipino women who smoke and reported e-cigarette use within the past 30 days conducted in November 2023.

	**To cut down smoking cigarettes** (n=262)	**To help quit smoking cigarettes** (n=262)	**To use when not allowed to smoke cigarettes** (n=262)	**Because vaping is less harmful than smoking to others** (n=255)	**Because I enjoy the flavor** (n=254)
	aOR (95% CI)	aOR (95% CI)	aOR (95% CI)	aOR (95% CI)	aOR (95% CI)
**Age**					
18 to 24 years old	ref	ref	Ref	Ref	ref
25-34 years old	0.85 (0.41-1.75)	1.28 (0.57-2.84)	0.83 (0.32-2.13)	0.91 (0.39-2.12)	1.24 (0.42-3.63)
≥ 35 years old	0.82 (0.36-1.85)	1.42 (0.55-3.68)	1.03 (0.34-3.09)	0.59 (0.20-1.78)	1.41 (0.43-4.69)
**Income (in PHP)**					
> 60,000	ref	ref	ref	ref	ref
60,000-299,999	1.43 (0.66-3.11)	1.41 (0.64-3.06)	1.17 (0.45-3.00)	1.30 (0.55-3.05)	0.70 (0.24-2.02)
≥ 300,000	3.31 (1.52-7.19)**	0.53 (0.20-1.37)	0.78 (0.28-2.18)	0.76 (0.28-2.07)	0.70 (0.23-2.16)
**E-cigarette device type**					
Reusable	ref	ref	ref	ref	ref
Disposable	0.99 (0.51-1.92)	1.24 (0.60-2.58)	0.30 (0.10-0.92)*	1.87 (0.87-4.00)	0.47 (0.15-1.48)
**Last e-cigarette flavor used**					
Tobacco/menthol	ref	ref	ref	ref	ref
All other flavors	1.18 (0.64-2.18)	0.73 (0.37-1.44)	1.08 (0.50-2.33)	1.62 (0.75-3.48)	1.81 (0.70-4.68)
**Frequency of e-cigarette use**					
Do not use weekly	1.41 (0.24-8.23)	1.73 (0.27-11.1)	1.04 (0.10-10.7)	empty	1.32 (0.13-13.5)
1-3 days per week	1.40 (0.76-2.57)	0.71 (0.35-1.42)	1.08 (0.50-2.35)	1.05 (0.50-2.18)	0.93 (0.39-2.45)
4-7 days per week	ref	ref	ref	ref	ref
**Smoking Dependence Level**					
Low	ref	ref	ref	ref	ref
Medium	1.03 (0.55-1.90)	1.29 (0.63-2.64)	2.33 (1.00-5.42)	0.93 (0.43-1.98)	0.54 (0.22-1.34)
High	0.38 (0.04-3.50)	3.74 (0.66-21.3)	1.35 (0.13-14.0)	0.86 (0.09-8.16)	empty
**Intention to quit smoking**					
Not interested/don't know	ref	ref	ref	ref	ref
Intend to quit within a year	2.28 (0.80-6.47)	5.37 (1.17-24.6)*	0.36 (0.13-1.00)	0.73 (0.25-2.15)	0.42 (0.12-1.33)
Intend to quit someday (> 1 year)	1.73 (0.52-5.73)	1.15 (0.19-7.00)	1.70 (0.56-5.14)	0.87 (0.25-3.04)	0.55 (0.14-2.13)

Note: aOR=adjusted odds ratio; CI=confidence interval; ref=reference group; PHP=Philippine peso; empty=there was no variation in selecting or not selecting the reason for use for this variable category and the model failed to provide a reliable estimate; the statistical software automatically dropped the observations that 'perfectly' predicted the outcome resulting in lower n's for some models
* p<0.05, **p<0.01, ***p<0.001; Significance level set at p<0.01 (or 0.05/5 comparisons)
